# Telemonitoring of Daily Activity and Symptom Behavior in Patients with COPD

**DOI:** 10.1155/2012/438736

**Published:** 2012-11-25

**Authors:** Monique Tabak, Miriam M. R. Vollenbroek-Hutten, Paul D. L. P. M. van der Valk, Job van der Palen, Thijs M. Tönis, Hermie J. Hermens

**Affiliations:** ^1^Telemedicine Group, Roessingh Research and Development, P.O. Box 310, 7500 AH Enschede, The Netherlands; ^2^Telemedicine Group, University of Twente, 7500 AE Enschede, The Netherlands; ^3^Department of Pulmonary Medicine, Medisch Spectrum Twente, 7500 KA Enschede, The Netherlands; ^4^Department of Research Methodology, Measurement and Data Analysis, University of Twente, 7500 AE Enschede, The Netherlands; ^5^Medical School Twente, Medisch Spectrum Twente, 7500 KA Enschede, The Netherlands

## Abstract

*Objectives*. This study investigated the activity behavior of patients with COPD in detail compared to asymptomatic controls, and the relationship between subjective and objective activities (awareness), and readiness to change activity behavior. *Methods*. Thirty-nine patients with COPD (66.0 years; FEV_1_% predicted: 44.9%) and 21 healthy controls (57.0 years) participated. Objective daily activity was assessed by accelerometry and expressed as *amount* of activity in counts per minute (cpm). Patients' baseline subjective activity and stage of change were assessed prior to measurements. *Results*. Mean daily activity in COPD patients was significantly lower compared to the healthy controls (864 ± 277 cpm versus 1162 ± 282 cpm, *P* < 0.001). COPD patients showed a temporary decrease in objective activities in the early afternoon. Objective and subjective activities were significantly moderately related and most patients (55.3%) were in the maintenance phase of the stages of change. *Conclusions*. COPD patients show a distinctive activity decrease in the early afternoon. COPD patients are moderately aware of their daily activity but regard themselves as physically active. Therefore, future telemedicine interventions might consider creating awareness of an active lifestyle and provide feedback that aims to increase and balance activity levels.

## 1. Introduction

Chronic Obstructive Pulmonary Disease (COPD) is a respiratory disease characterized by the progressive development of airflow limitation in the lungs, causing primarily shortness of breath (dyspnea) and diminishing physical exertion capabilities [1, 2]. Symptomatic patients with COPD are dyspnoeic even when they perform normal daily activities, which leads to inactivity and, subsequently, to physical deconditioning [1]. A vicious cycle develops that greatly affects quality of life [1, 2]. Regular physical activity in COPD has been associated with a reduction of the risk of hospital (re)admission [3–5], increase of life expectancy [6], and slowing the rate of decline in lung function [7]. The importance of an active lifestyle is underlined by several studies that showed the inactivity of COPD patients compared to healthy individuals, for example, [8–15]. This decrease in activity levels is not caused solely by airflow limitation, and other factors like dynamic hyperinflation and systemic inflammation seem to play an important role as well [16–18].

In addition to increasing activity levels, a more equally distributed daily activity pattern is assumed to improve patients' well-being. In daily care healthcare professionals therefore advise their patients to plan their days and weeks carefully to use their energy efficiently, spreading chores and alternating heavy activities with light activities over the day, but these advices can be difficult to apply in daily life. Unbalanced activity patterns have been found in patients with chronic low back pain (CLBP) and chronic fatigue syndrome (CFS), showing reduced levels of activity in the afternoon and evening compared to healthy controls [19, 20]. Previous research already showed that telemedicine applications can positively influence the daily activity behavior for patients with CLBP [21] and CFS [22]. These telemedicine interventions measure the activity behavior by an activity sensor and provide personalized feedback messages with advice on how to improve the measured activity on a smartphone. Patients with COPD might also benefit from such applications, but detailed monitoring information about the activity behavior of COPD patients during the day is not yet available. This should first be investigated in detail, before these interventions can be designed. Moreover, the information about whether symptoms influence this activity during the day is not yet available. This together makes it difficult to determine where to concentrate on in (future) treatment.

A prerequisite for treatment to change the activity behavior—by increasing and balancing activity patterns—is awareness. Patients need to be aware of their activity behavior, otherwise treatment is unlikely to be effective [23, 24]. The ability to understand the activity behavior, the relationship with daily symptoms, and the willingness of the patients to change is important for the success of any treatment aiming to improve activity behavior of COPD patients.

Therefore, this study aimed to investigate the activity behavior of patients with moderate to severe COPD during the day in comparison with asymptomatic controls. A triaxial accelerometer was used to measure activity objectively. The second aim of this study was to investigate how symptoms change during the day and whether they are related to the amount of activity during the day. A smartphone was used to score symptoms during the day. Finally, we investigated the relationship between subjectively and objectively measured activity to assess COPD patients' awareness and their readiness to change activity behavior based on the stages of change [24].

## 2. Methods

### 2.1. Subjects

Thirty-nine patients with COPD (66.0 ± 8 years; 23 male, 16 female) with a clinical diagnosis of stable COPD, that is, no infection or exacerbation in the 4 weeks prior to measurement, were recruited at Medisch Spectrum Twente hospital at Enschede, the Netherlands. A postbronchodilator spirometry recording using ERS standards was used to confirm patients' diagnosis, to measure the forced expiratory volume in one second percent predicted (FEV_1_% predicted) and to classify the patients in one of the GOLD stages [2]. Other inclusion criteria were presence of dyspnea, current or former smoker, ability to read and speak Dutch, and age between 40 and 80 years. Exclusion criteria were a rapidly declining clinical course, use of a wheelchair, use of long-term oxygen therapy, a history of asthma, any medical condition impairing the activities of daily life, serious psychiatric comorbidity, and participation in a COPD rehabilitation program in the past 3 months.

In addition, 21 asymptomatic controls without (57.0 ± 4.5 years; 8 male, 13 female) a history of asthma or COPD, or any medical condition that impairs normal daily activities, were recruited from staff, their relatives, and through advertisements. The same exclusion criteria applied for controls. All participants gave their informed consent. 

### 2.2. Daily Measures

Objective daily activity was assessed using the MTx-W sensor (Xsens Technologies B.V., P.O. Box  559, 7500 AN Enschede, the Netherlands), which measures 3D acceleration (90 × 45 × 17 mm, 77 g). The output measure is calculated following the method of Bouten et al., [25] which is highly related to energy expenditure [25, 26]. The accelerometer data (output frequency: 100 Hz) was band-pass filtered using a 4th order Butterworth filter, with cut-off frequencies of 0.11 Hz and 20 Hz. The absolute accelerometer signals were integrated over 60 seconds and summed over the three axes, and the final output was expressed in activity counts per minute (cpm). For each measurement direction, sensitivity is set at 1000 counts/min, corresponding to an acceleration of 1 g. The sensor system communicated wirelessly with a smartphone (HTC P3600/3700) by Bluetooth, which stored the data on the storage card.

**Figure 1:**
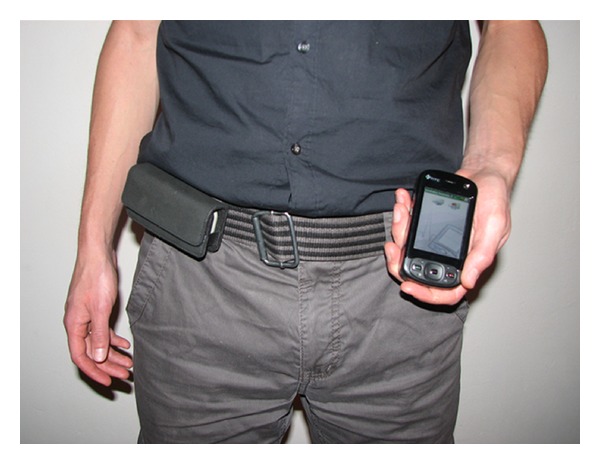
participant wearing the sensor on the belt and holding the smartphone.

Both the activity sensor and smartphone were worn on the subject's belt (Figure 1). Daily activity was assessed for four consecutive days from waking to 22:00 h. Previous studies have shown that two days of measurement are required to reliably (intraclass correlation coefficient > 0.8) measure physical activity in GOLD II patients [14] and three days in GOLD III patients [27]. Sundays were found to be a day of reduced activity and variability was higher in less severe COPD patients [28]. Therefore, a measurement period of four days was chosen in the present study, excluding Sundays. Participants were asked to continue the routine of their daily life during measurement.

Using the smartphone, the COPD patients answered questions at fixed time intervals (13:00 h, 17:00 h, and 20:00 h) during the day about self-perceived activity performance—to assess awareness—and dyspnea and fatigue levels by means of visual analogue scales (VAS).

### 2.3. Subjective Activity and Stage of Change

The general self-perceived amount of activity of the COPD patients was measured using the Baecke Physical Activity Questionnaire (BPAQ) to assess activity awareness. The BPAQ covers questions about work activities, sports, and leisure-time activities (range: 3–15). The stage of change questionnaire was used to assess the patients' motivation to change their activity behavior, according to the Transtheoretical model [24]. This defines five principle stages of change: precontemplation, contemplation, preparation, action, and maintenance. The questionnaires were administered before start of the measurement.

### 2.4. Data Analysis

Mean activity per hour for each subject was calculated and line graphs were made that show the average activity per hour. Only those hours between 8:00 h and 20:00 h, for which at least 50% of the data for that particular hour was available, were included in the analysis. Data points could be missing due to the following: (1) the device was switched on/off in the middle of an hour (e.g., at waking, or when swimming/showering), or (2) connection/battery problems. Three day parts were evaluated in the analysis: morning (8:00 h to 13:00 h), afternoon (13:00 h to 17:00 h), and evening (17:00 h to 20:00 h).

For the VAS questions, the mean VAS score for each subject per day part was calculated, and line graphs were made that show the average VAS scores per day part.

### 2.5. Statistical Analysis

The Statistical Package for the Social Sciences (SPSS, 18.0) was used for statistical analyses. The results are described in terms of mean (SD) or percentage. For all parameters, the mean of the measurement days per subject was used for analysis. Data on activity was normally distributed and comparison between the two groups in mean daily activity and activity levels were performed using the independent *t*-test. When comparing more than two categories, analysis of variance (ANOVA) with Sidak post hoc test was used or the Kruskal-Wallis test with post hoc Mann-Whitney *U* tests with Holm-Bonferroni correction, as appropriate.

The Pearson product-moment correlation coefficient was calculated to evaluate the relationships of the objectively measured daily activity with continuous variables (e.g., age) or subjective daily activity (BPAQ). For comparing two categorical variables (such as gender with work status), Pearson Chi-square was used.

For the subject characteristics we investigated possible significant differences between the healthy group and the COPD group and possible significant correlations with mean daily activity. Confounding factors werecontrolledby using a univariate linear regression model. Effect modification of the relationship between group (COPD/healthy control) and activity was a priori suspected for age and work status. This was formally tested by including interaction terms in the regression models. In case of effect modification, data is presented in subgroups.

## 3. Results

### 3.1. Participants

Characteristics of the patients with COPD are listed in Table 1.

The healthy control group (*n* = 21)  had a mean age of 57.0 ± 4.5 years and consisted of 8 males and 13 females with a mean BMI of 26.9 ± 3.6. In this group, 52.4% were employed, and 47.6%  were unemployed. There was no significant difference found for gender or BMI between the two groups. Age and work status differed significantly between the two groups (resp, *P* < 0.001 and *P* = 0.006).

### 3.2. Mean Daily Activity

Mean daily activity—the amount of activity per day—in COPD patients was significantly lower compared to the healthy controls, 864 ± 277 cpm versus 1162 ± 282 cpm, *P* < 0.001. Taking both groups together, mean daily activity was not significantly related to work status (*P* = 0.067), but significantly related to age (*r* = −0.33, *P* = 0.009). In a linear regression model using mean daily activity (in cpm) as dependent variable, and group (COPD/control) and age as independent variables, age did not significantly contribute to the model (*P* = 0.356). There also was no age-group interaction (*P* = 0.617). In the same manner, work status did not significantly contribute (*P* = 0.506) as confounder, but there was a work-group interaction (*P* = 0.018) meaning that work status does not influence the activity behavior in the total group; however, within both groups the relationship between work status and activity behavior differs significantly. Therefore, data will be presented separately for those with and without work, respectively.

### 3.3. Activity Behavior

Table 2 presents the mean activity for both the COPD group and the asymptomatic control group, by day, morning, afternoon, and evening, stratified for work status. Unemployed patients with COPD were significantly less active compared to the unemployed healthy controls over the entire day, as well as for all day parts. However, employed patients with COPD were equally active compared to the employed healthy controls over the entire day, as well as for all day parts.

For unemployed patients with COPD the difference in mean activity *between* day parts was significant for morning-evening (*P* < 0.001) and afternoon-evening (*P* = 0.012), and reached borderline significance for morning-afternoon (*P* = 0.056). The mean activity for the three day parts was not significantly different for employed patients with COPD (*P* = 0.336), employed controls (*P* = 0.074), and unemployed controls (*P* = 0.246).

**Figure 2:**
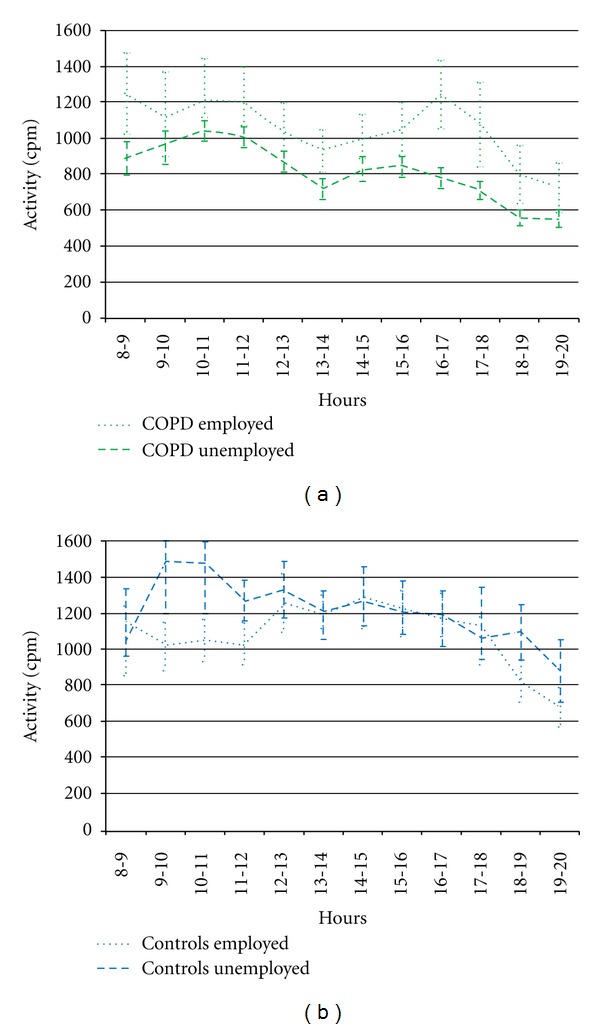
The daily activity pattern in mean activity per hour (cpm) for both the COPD group and the healthy control group with standard errors of the means.

Figure 2 shows the mean activity per hour—the daily activity pattern—for the patients with COPD and asymptomatic controls, stratified for employment status. The COPD group showed a dip of lower activity in their daily activity pattern in the early afternoon. This dip occurs in the activity pattern of COPD patients both with and without work. This dip does not occur in the control group.

### 3.4. Relation between Physical Activity and COPD Symptoms during the Day

Assessed by VAS questions on the smartphone, dyspnea levels remained constant during the day (VAS morning: 2.7 ± 1.8, afternoon: 2.9 ± 1.6, evening: 2.9 ± 1.8) in COPD patients. Fatigue was highest in the afternoon (VAS morning: 3.0 ± 1.8, afternoon: 3.6 ± 1.9, evening: 3.0 ± 2.0), but this difference was not statistically significant.

**Figure 3:**
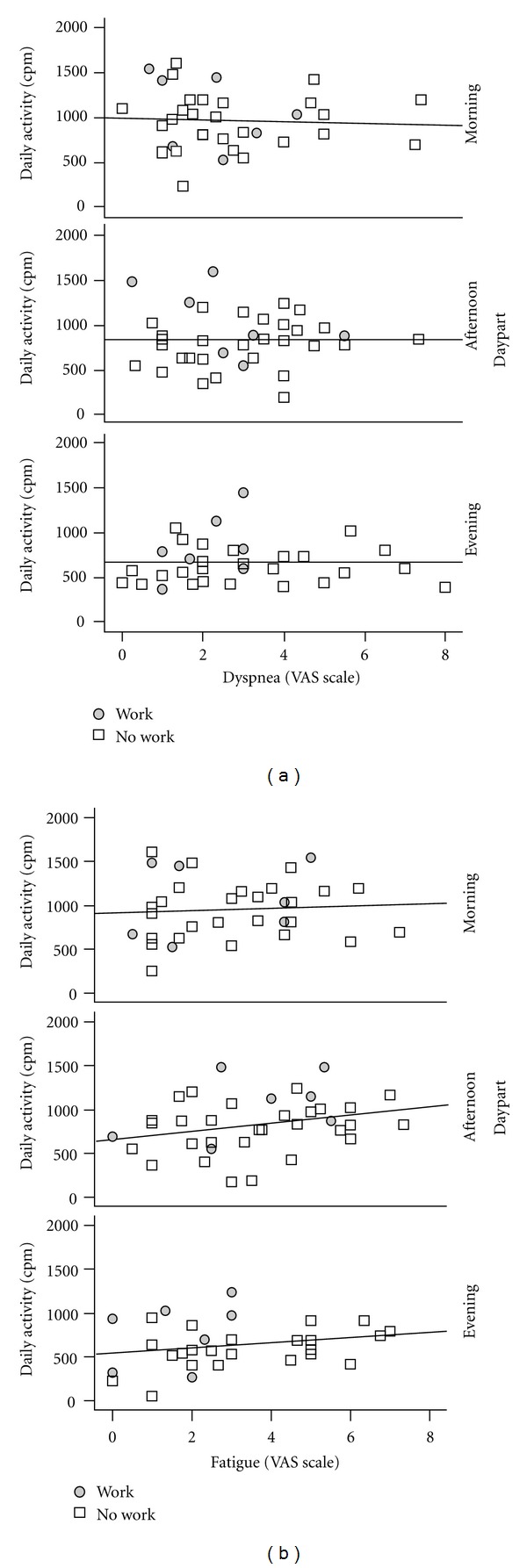
Scatter plots per day part for objectively measured activity (in cpm) (*y*-axis) and symptom scores (VAS score, *x*-axis), with linear trend line. On the left this is shown per day part for dyspnea, on the right for fatigue. Each dot represents the mean of one patient, stratified for employment.

To investigate the relation between activity and symptoms during the day, correlations of dyspnea and fatigue with objectively measured activity per day part were investigated (Figure 3). Both fatigue and dyspnea levels were not significantly related to activity during the day.

### 3.5. Activity Awareness and Stages of Change

To investigate the activity awareness of COPD patients during the day, VAS questions to rate the perceived activity were asked thrice daily. This self-perceived activity was moderately correlated to the objectively measured activity (in cpm). Morning: *r* = 0.54, *P* = 0.001; afternoon: *r* = 0.57, *P* < 0.001; evening: *r* = 0.56, *P* = 0.001.

**Figure 4:**
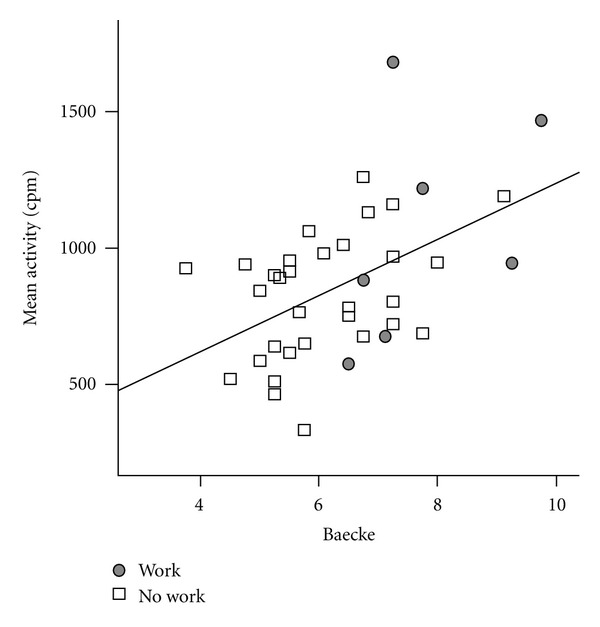
scatter plot of COPD patients for subjective activity (BPAQ) (*y*-axis) and objective activity (accelerometer) (*x*-axis), stratified for employment with linear trend line.

Also, the BPAQ was measured prior to measurement, to investigate general activity awareness. COPD patients had a mean subjective activity of 6.4 ± 1.3, which was moderately correlated to objectively measured activity: *r* = 0.49, *P* = 0.002. In Figure 4 this is shown, stratified for employment status.

With regard to physical activity, the majority (55.3%) of the COPD patients were in the maintenance phase of the stages of change model (precontemplation: *n* = 0, contemplation: *n* = 3, preparation: *n* = 10, action: *n* = 4, maintenance: *n* = 21, 1 missing). Using Kruskal-Wallis, the stages were not significantly related to objective activity levels (employed: *P* = 0.317, unemployed: *P* = 0.174).

## 4. Discussion

The aim of this study was to investigate the activity behavior of patients with moderate to severe COPD during the day in comparison with asymptomatic controls and the relationship with symptoms during the day. Furthermore, the goal of this study was to investigate whether COPD patients are aware of their own daily activity and ready to change their activity behavior. This can provide a starting point for designing new telemedicine interventions that aim to improve activity behavior for COPD patients.

The results of this study show that COPD patients are significantly less active than controls. These results correspond to findings in previous studies, showing reduced amounts of activity in COPD patients [10–15]. In our study, there was a large age difference reported between groups, as well as difference in employment status. However, only employment status affected activity levels in both groups, therefore results were presented in subgroups. In literature, work status of the patients was not reported [11–13, 15], only unemployed participants were included [14], or no relationship between work status and activity was found [10]. Based on our results, we expect that work status could importantly influence results on activity behavior. Our study shows that unemployed COPD patients are almost 35% less active than unemployed controls, while the activity level of employed patients and employed controls is approximately the same. This could be due to the healthy worker effect; an individual must be relatively healthy to be employable in a workforce. Patients that are unemployed are not working due to their ill health; they are not able to function on an activity level needed for their job. This is emphasized by the fact that COPD patients in general have a lower socioeconomic status, and consequently more physically demanding jobs. Unemployed healthy people are not restricted anymore by their day time jobs and can now plan and do fun visits and activities, thereby being more active.

Both employed patients and unemployed patients show a temporarily decrease in activity in the early afternoon. This suggests that they perform too many activities in the morning, resulting in an activity relapse in the afternoon. Activity again increases in the late afternoon, especially in employed COPD patients. Visual inspection of the daily activity patterns of each individual patient with COPD shows that this trend observed for the average population is shown in the majority of the patients. These findings underpin the professionals' advice to their patients to use their energy efficiently during the day. Hecht et al. showed that in very severe COPD patients using LTOT a sharp decrease in activity is present in the early afternoon, but activityonly shows a small recovery afterwards in the evening [29]. Telemedicine interventions could balance this activity pattern [21], but whether a more distributed daily activity pattern indeed improves physical health status and well-being of COPD patients should be investigated in future studies.

Our results show that the amount of activity per day part of unemployed patients is significantly below that of unemployed controls for all day parts, while this is not the case for the employed. This is differentfromother chronic patient groups, where normal levels of activity were found in the morning, and only reduced levels of activity in the afternoon and evening [19, 20]. These studies suggested that these decreased activity levels could correspond to increased pain or fatigue intensities during the day, but this was not yet investigated. In the present study, we used a smartphone to rate symptom levels retrospectively on a visual analogue scale three times a day. Our study shows that the relationship of symptoms and activity during the day was not clearly present, and there seem to be different factors that determine patients' distinctive activity pattern. This could require further investigation in future studies.

Regarding the last research objective, we investigated the relationship between subjective and objective measured activity to assess COPD patients' awareness and their readiness to change their activity behavior based on the stages of change. Patients should be aware of their amount of daily activity and motivated to change their behavior, otherwise a treatment is unlikely to be effective in the long run, as indicated by the Transtheoretical model [24]. By using a subjective measurement such as the BPAQ the patient with COPD can be assessed on the awareness of his or her daily activity [30]. Our results show that the objective daily activity and subjective daily activity assessed by the BPAQ are significantly related for patients with COPD (*r* = 0.49), which is a bit less than reported in literature for healthy controls (*r* = 0.66), but higher than for chronic low back pain patients (*r* = −0.27) [31]. In other words, patients seem fairly aware of their amount of activity, which is an important finding for treatment. This relationship between objective and subjective activity is also present for the different day parts. Furthermore, the majority of the included patients were in the maintenance phase of the stages of change model, meaning that they regard themselves as being regularly physically active for an extended period of time and think that their current activity behavior is fine. The data indeed shows that patients in the maintenance phase were not more active compared to patients in other stages. These are very important findings; although patients seem to be aware of their daily activity, they feel fine with the current situation and do not have the intention to change their present activity behavior. Future interventions might consider focusing on the importance of an active lifestyle, and motivating patients to change their behavior.

We used a validated method for measuring activity behavior; however our study had some limitations. The wireless Bluetooth connection between the sensor and smartphone was a drain on the batteries of both devices meaning that the devices would often run out of power after 12 hours of operation. Besides, charging of the sensors was experienced to be difficult, resulting in not fully charged sensors and thus less lengthy measurement days. As a consequence, the hours after 20:00 h were excluded from analysis. Further advancements in the field of wireless sensor technology and mobile devices should overcome these issues in future telemedicine treatment. Furthermore, we did not include a functional capacity measurement, such as the 6-minute walking test. This might be a useful outcome measure in the evaluation of future (telemedicine) treatments; can the use of a telemedicine system change activity behavior and, consequently, can it improve patients' functional capacity?

## 5. Implications

Our study used telemonitoring to assess the activity and symptom behavior during the day, using a 3D accelerometer for activity monitoring and a smartphone for monitoring symptom levels. Telemonitoring of activity and symptoms can be a valuable tool in daily practice, for professionals and patients to monitor patients' progress and well-being, both in primary and secondary care. Moreover, telemonitoring provides new information and insights from daily life and supports evidence-based treatment. This study provides a first insight in the activity behavior in more detail, and its relations with symptoms levels during the day. We can conclude that COPD patients, especially unemployed, have a low and imbalanced activity pattern compared to healthy controls. Therefore, we should aim to restore activity levels and it might be considered to pay special attention to the distribution of activities over the day. Furthermore, to let treatment be effective, treatment should make patients aware of their activity behavior and the importance of an active lifestyle.

Based on the outcomes of telemonitoring studies, we can start designing new and effective treatment methods for improving activity behavior. Also, telemonitoring can be integrated with telemedicine applications, like online exercise programs or ambulant personalized feedback, to improve activity behavior. This feedback could raise awareness of the activity behavior and motivate patients to change. Previous studies already showed that pedometer feedback could be used to increase physical activity levels [32–34]; however research into effective feedback strategies is still in its infancy [23].

## Figures and Tables

**Table 1:** Patients' characteristics and health status.

Characteristics	*n*	Mean ± SD	Frequency	Percentage (%)
Age (years)	39	66.0 ± 8.1		
Gender	39			
Male			23	59
Female			16	41
FEV_1_% predicted	39	44.9 ± 15.5		
GOLD stage	39			
II			13	46.2
III			18	33.3
IV			8	20.5
Smoking	38			
Current smoker			10	25.6
Former smoker			28	71.8
MRC	38			
1			7	17.9
2			12	30.8
3			14	35.9
4			3	7.7
5			2	5.1
BMI (kg/m^2^)	38	26.7 ± 4.9		
Work status	39			
Employed			7	17.9
Unemployed			32	82.1

Abbreviations: FEV_1_% predicted: forced expiratory volume in 1 second percent predicted, GOLD: Global Initiative for Chronic Obstructive Lung Disease, MRC: medical research council dyspnea scale, BMI: Body Mass Index.

**Table 2:** Mean daily activity in counts per minute in COPD patients (*n* = 39) and controls (*n* = 21), stratified for employment status (COPD: employed (*n* = 7)/unemployed (*n* = 32), controls: employed (*n* = 11)/unemployed (*n* = 10)).

	COPD patients	Controls	95% CI	*P* value
Day				
Employed	1066 ± 409	1091 ± 187	−323; 274	*P* = 0.865
Unemployed	820 ± 225	1241 ± 352	−611; − 231	*P* < 0.001
Morning (8–13 h)				
Employed	1165 ± 545	1087 ± 307	−344; 501	*P* = 0.699
Unemployed	958 ± 281	1382 ± 511	−682; − 165	*P* = 0.002
Afternoon (13–17 h)				
Employed	1088 ± 369	1235 ± 304	−486; 192	*P* = 0.371
Unemployed	804 ± 260	1241 ± 301	−635; − 240	*P* < 0.001
Evening (17–20 h)				
Employed	830 ± 355	911 ± 343	−438; 275	*P* = 0.634
Unemployed	615 ± 212	1078 ± 343	−647; − 279	*P* < 0.001

Data is presented as mean ± SD. 95% CI: 95% confidence interval of the difference.
